# Supplementary material for: A RAD51 assay feasible in routine tumor samples calls PARP inhibitor response beyond BRCA mutation
Source: EMBO Mol Med. 2018 Oct 30;10(12):e9172. doi: 10.15252/emmm.201809172 (PMC6284440; doi:10.15252/emmm.201809172)
Supplement: Supplementary file 1 — Appendix [file EMMM-10-e9172-s001.pdf]

APPENDIX

Table of contents

1) Appendix Table S1
2) Legends to Appendix Figures
3) Appendix Figure S1
4) Appendix Figure S2
5) Appendix Figure S3
6) Appendix Figure S4
7) Appendix Figure S5

Legends to Appendix Figures

Appendix Figure S1. Immunofluorescence staining of BRCA1 nuclear foci in PDXs. Cells in the S/G2-phase (geminin-positive, green) with or without BRCA1 nuclear foci (red) are shown for four PDXs models from cohort-1: two PARPi-resistant PDXs (PDX098 and PDX060) and two PARPi-sensitive PDXs (PDX197 and PDX093). Scale bars: 10 µm.

Appendix Figure S2. Immunofluorescence staining of RAD51 nuclear foci in PDXs. Cells in the S/G2-phase (geminin-positive, green) with or without RAD51nuclear foci (red) are shown for four PDXs models from cohort-1: two PARPi-resistant PDXs (PDX098 and PDX060) and two PARPi-sensitive PDXs (PDX197 and PDX093). Scale bars: 10 µm.

Appendix Figure S3. Restoration of BRCA1 foci formation in PARPi acquired-resistant PDXs. Immunofluorescence staining of BRCA1 foci in PARPi-treated tumors from STG201, PDX302 and the corresponding PARPi acquired-resistant models (STG201OR and PDX302OR). Cells in S/G2-phase (green) with or without BRCA1 foci (red) are shown. Nuclei were visualized with DAPI (blue). Scale bars: 10 µm.

Appendix Figure S4. Restoration of RAD51 foci formation in PARPi acquired-resistant PDXs. Immunofluorescence staining of RAD51 foci in PARPi-treated tumors from STG201, PDX302 and the corresponding PARPi acquired-resistant models (STG201OR and PDX302OR). Cells in S/G2-phase (green) with or without RAD51 foci (red) are shown. Nuclei were visualized with DAPI (blue). Scale bars: 10 µm.

Appendix Figure S5. Analysis of RAD51 foci cut-off. Scatter plots showing the number of RAD51 foci in each individual geminin-positive cell (100 cells for each model) in **A**) vehicle- or **B**) olaparib-treated samples from six PARPi-resistant and three PARPi-sensitive PDXs models from cohort-1. The dashed line indicates the cut-off of 5 RAD51 foci-per-nucleus. **C**) Forest plots and Odd's ratio analysis and **D**) accuracy and specificity of the RAD51 score using different cut-offs from 1 to 10 foci per nucleus.

Appendix Table S1: Initial and end tumor volumes of vehicle- and olaparib-treated tumors for PDXs cohort-1.

	Vehicle							Olaparib								
Sample ID	Days	Mean Vi	SEM Vi	Mean Vf	SEM Vf	N	TVC (%)	Days	Mean Vi	SEM Vi	Mean Vf	SEM Vf	N	TVC (%)	p value Vi	p value TVC
PDX302OR	22	81.37	2.99	360.35	22.94	3	343	21	90.61	8.21	487.45	133.32	3	438	0.35	0.64
STG201OR	21	89.4	22.89	409.59	99.56	5	358	21	86.60	15.18	389.09	92.96	4	349	0.93	0.55
PDX98	21	241.17	52.90	913.26	208.25	5	279	21	190.33	32.37	698.20	65.85	7	267	0.41	0.80
STG139	21	205.67	34.26	642.34	100.58	7	212	21	212.98	29.31	772.58	139.57	8	263	0.87	0.52
PDX137	20	148.35	18.47	818.76	108.30	5	452	20	204.70	27.24	739.60	102.69	8	261	0.16	<0.0001
PDX93OR	22	152.09	56.37	446.11	133.37	6	193	21	130.45	23.59	446.22	123.24	6	242	0.73	0.48
PDX102	20	252.71	70.01	962.57	226.44	7	281	20	233.38	22.52	515.47	72.96	12	121	0.75	0.10
PDX44	20	206.20	42.35	554.29	135.56	7	169	20	211.79	39.28	499.54	92.52	7	136	0.92	0.34
PDX156	20	112.76	18.07	515.25	181.89	6	357	20	160.52	26.12	283.44	80.27	4	77	0.16	0.13
PDX291	21	150.67	53.51	208.67	44.56	3	38	21	182.15	56.34	337.07	123.98	3	85	0.71	0.48
PDX94	21	273.23	66.72	910.23	232.84	11	233	21	218.95	54.49	463.76	97.02	7	112	0.57	0.67
PDX60	22	198.75	22.07	874.50	266.26	4	340	22	248.14	41.95	430.12	126.03	6	73	0.40	0.06
PDX288	19	178.6	51.70	401.00	128.34	5	125	19	236.69	13.27	379.61	62.02	9	60	0.19	0.18
PDX270	21	205.53	73.94	458.05	221.39	4	123	20	243.19	18.74	432.25	105.89	5	78	0.58	0.19
PDX302	12	163.00	23.03	770.25	177.03	4	373	64	148.21	25.02	17.12	17.12	7	-88	0.70	<0.0001
STG201	21	230.80	51.78	561.80	158.26	5	143	39	232.10	30.66	77.19	61.98	9	-67	0.98	<0.0001
PDX197	21	277.46	35.32	1917.76	516.71	6	591	20	302.36	66.36	0.00	0.00	6	-100	0.29	0.03
PDX93	22	120.97	25.66	338.36	38.04	10	180	68	179.07	24.51	0.00	0.00	6	-100	0.15	0.01

Vi: Initial Volume
Vf: Final Volume
TVC: Tumor Volume Change
Statistical tests were performed using unpaired two-tailed t-test for Vi and TVC (%) comparison between vehicle- and olaparib-treated tumors in each PDX model.

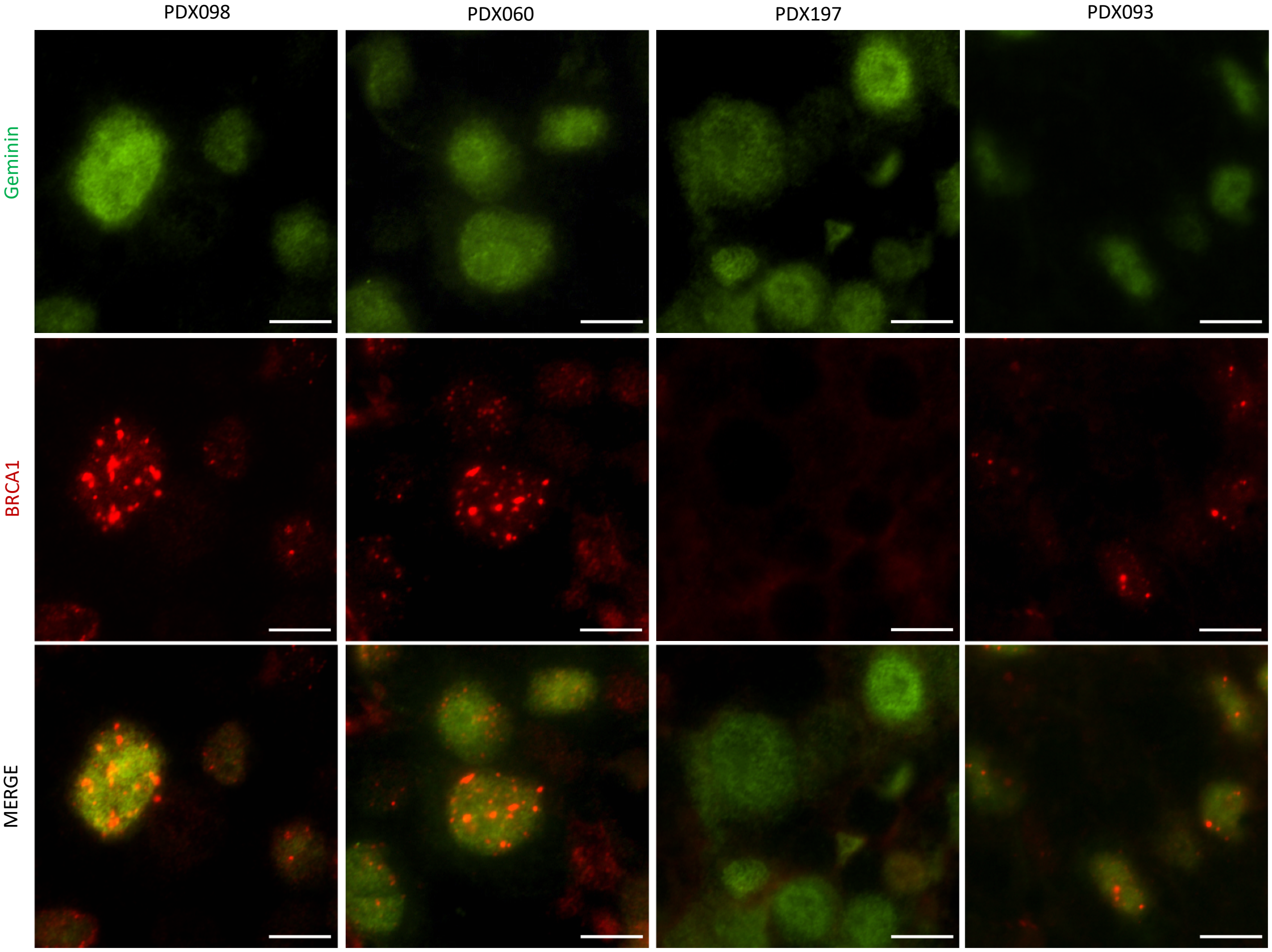

Appendix Figure S1

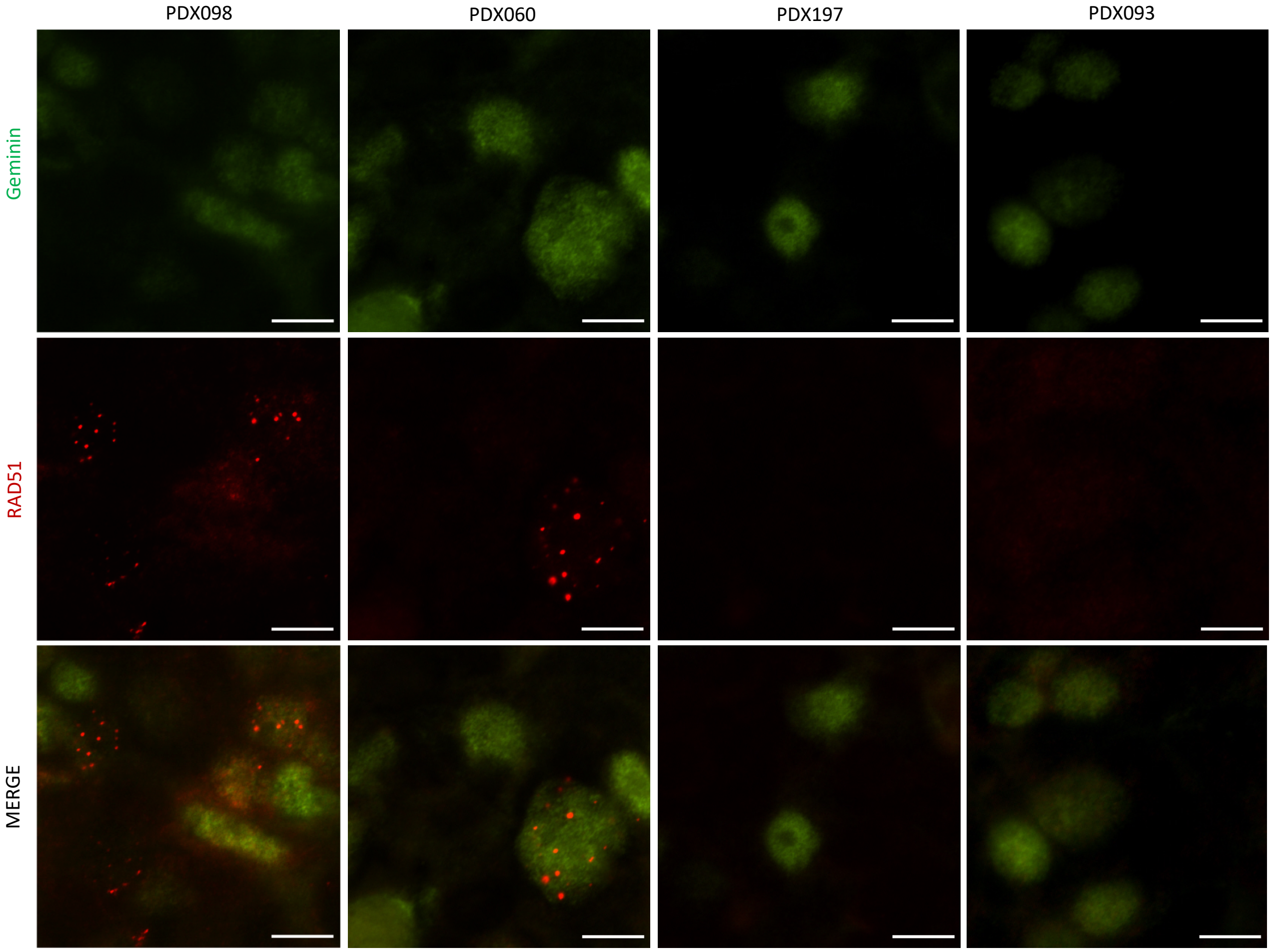

Appendix Figure S2

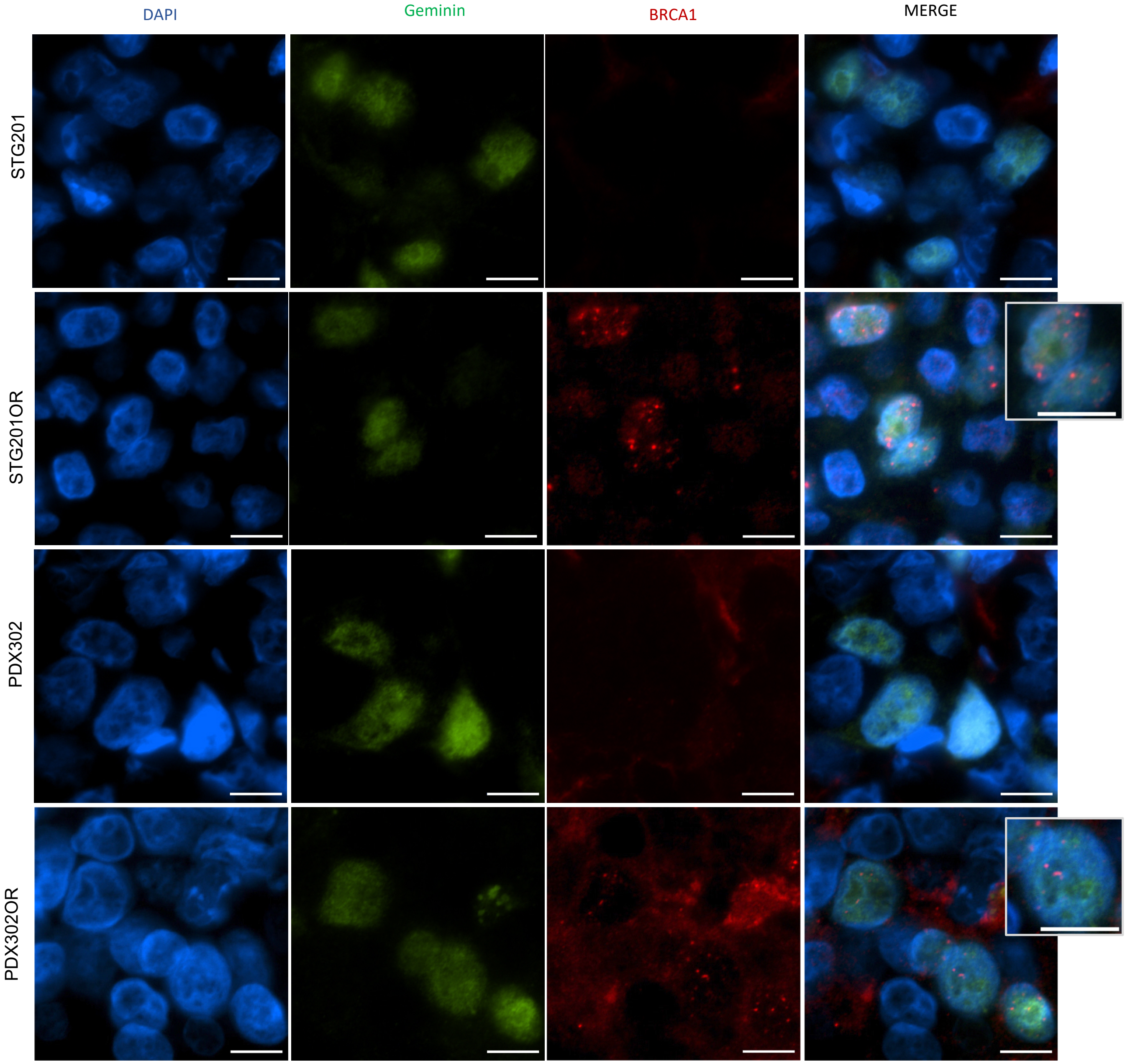

Appendix Figure S3

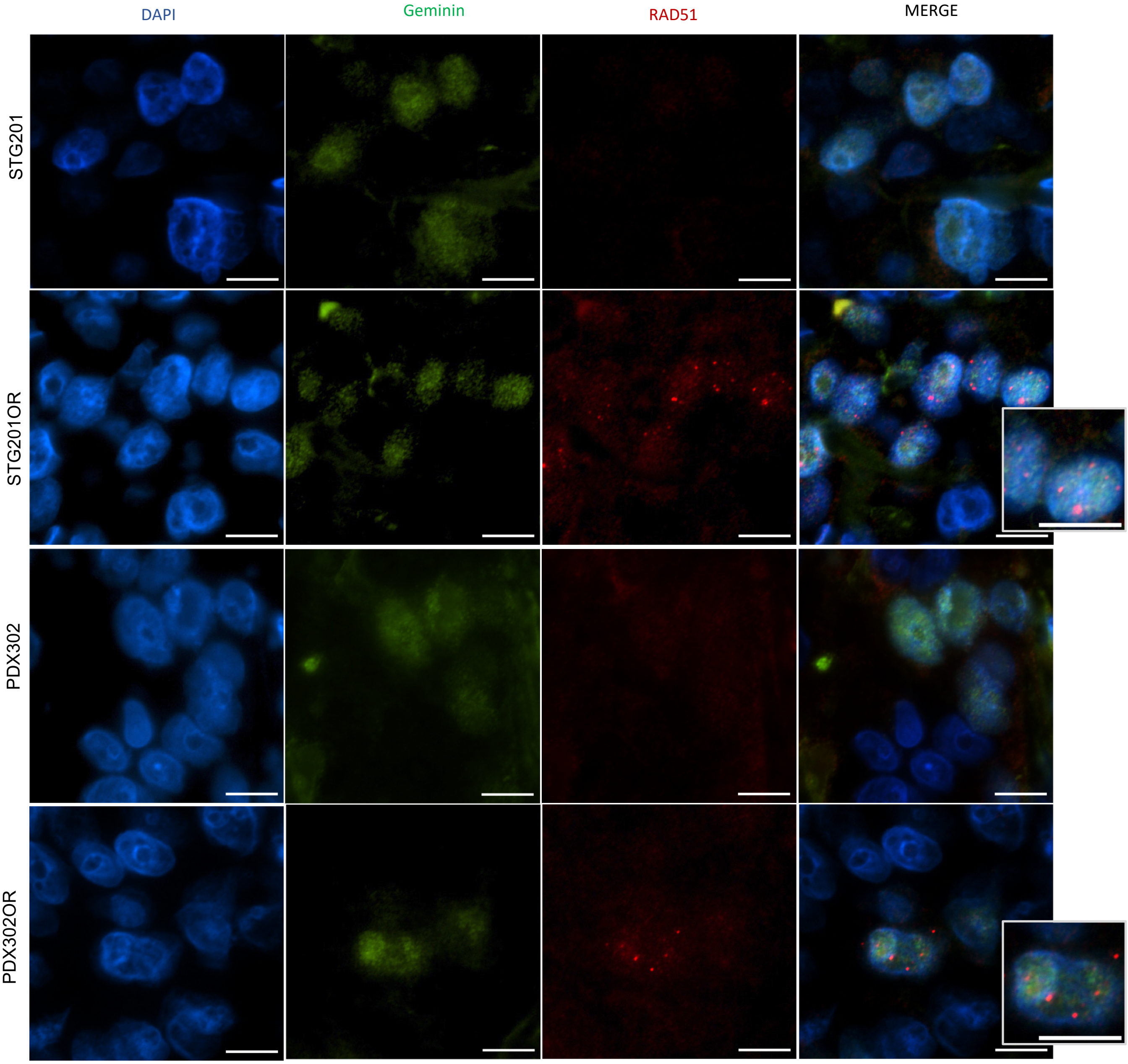

Appendix Figure S4

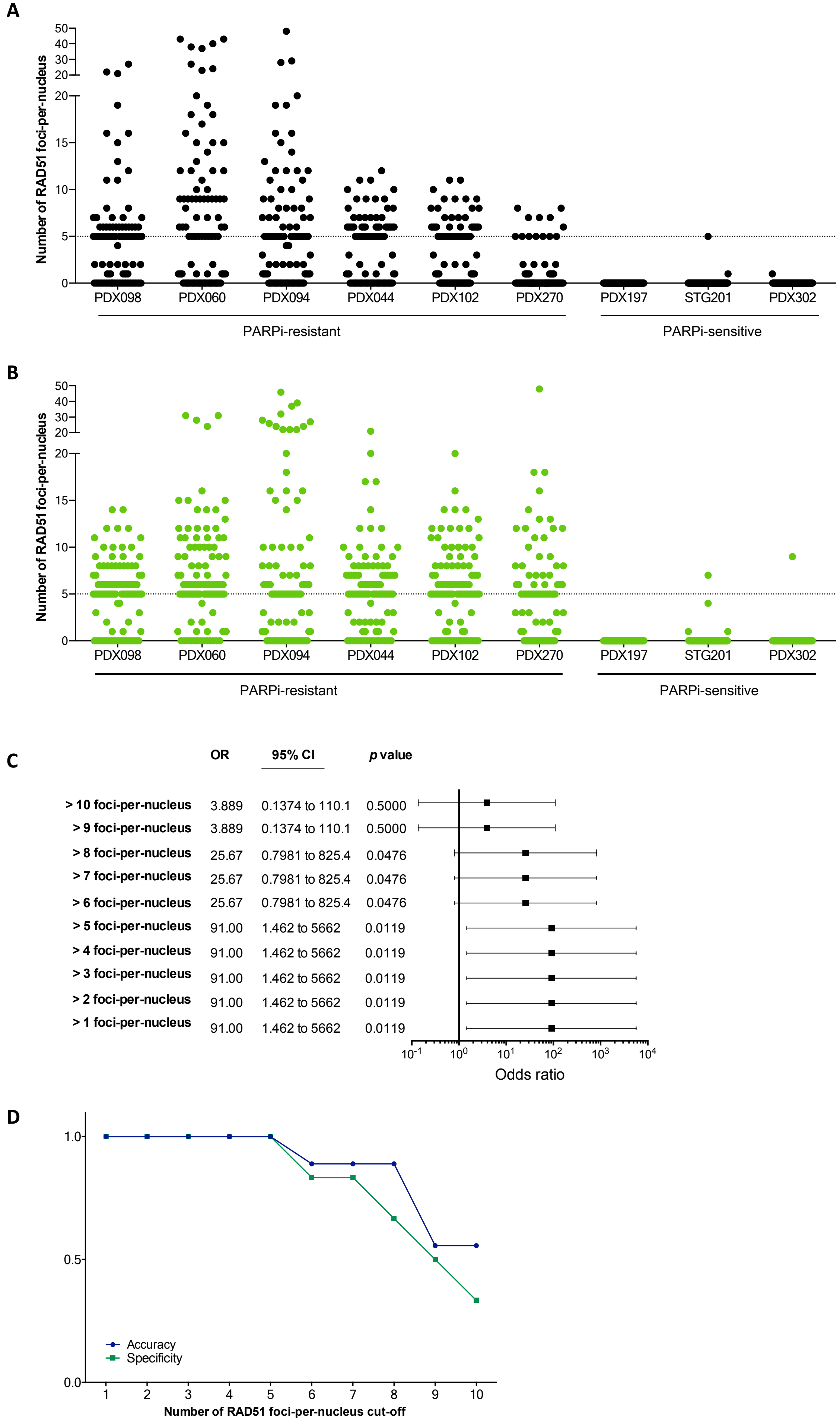

Appendix Figure S5
