# Supplementary material for: A RAD51 assay feasible in routine tumor samples calls PARP inhibitor response beyond BRCA mutation
Source: EMBO Mol Med. 2018 Oct 30;10(12):e9172. doi: 10.15252/emmm.201809172 (PMC6284440; doi:10.15252/emmm.201809172)
Supplement: Supplementary file 2 — Expanded View Figures PDF [file EMMM-10-e9172-s002.pdf]

Expanded View Figures

Figure EV1. Analysis of *PALB2* mutation in PDX093; RAD51 assay scoring and validation.

A Sanger sequencing reverse plots of DNA and RNA from PDX093 showing 1 nt (T) insertion in one *PALB2* allele (*PALB2* c.886dupA).
B Flow diagram of the RAD51 scoring criteria and quality controls.
C Quantification of geminin-positive cells with RAD51 foci (Mann–Whitney *U*-test) following treatment with vehicle and olaparib in PARPi-sensitive (CR/PR) versus PARPi-resistant (PD) PDXs.
D Pearson correlation between the RAD51 score (percentage of RAD51 (+)/Geminin (+) cells, assessed in untreated FFPE tumor samples) and the percentage of tumor volume change in olaparib-treated tumors from PDX cohort-1. Each dot represents one PDX model. Error bars indicate SEM from independent tumors ($n \geq 3$) treated with olaparib.
E Quantification of geminin-positive cells (paired *t*-test in vehicle- versus olaparib-treated tumors; unpaired *t*-test in CR/PR versus PD tumors) following treatment with vehicle and olaparib in PARPi-sensitive (CR/PR) versus PARPi-resistant (PD) PDXs.
F Spearman correlation of the percentage of RAD51 (+)/Geminin (+) cells in PDX cohort-1 assessed with two different antibodies against RAD51 both in vehicle- and olaparib-treated PDX samples.

© 2018 The Authors

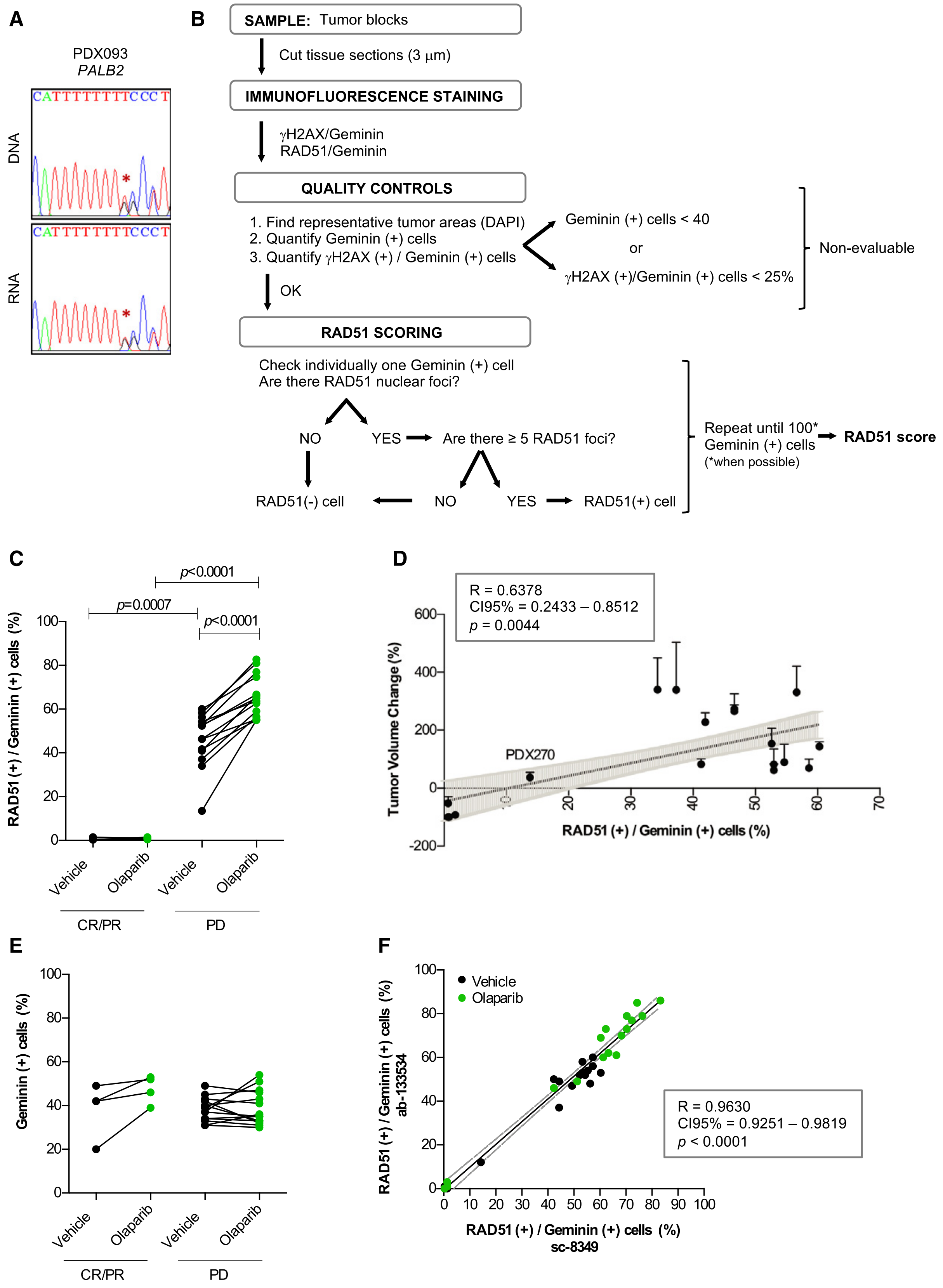

Figure EV1.

© 2018 The Authors
