# Supplementary figures and images for: A RAD51 assay feasible in routine tumor samples calls PARP inhibitor response beyond BRCA mutation
Source: EMBO Mol Med. 2018 Oct 30;10(12):e9172. doi: 10.15252/emmm.201809172 (PMC6284440; doi:10.15252/emmm.201809172)

Figure 2B

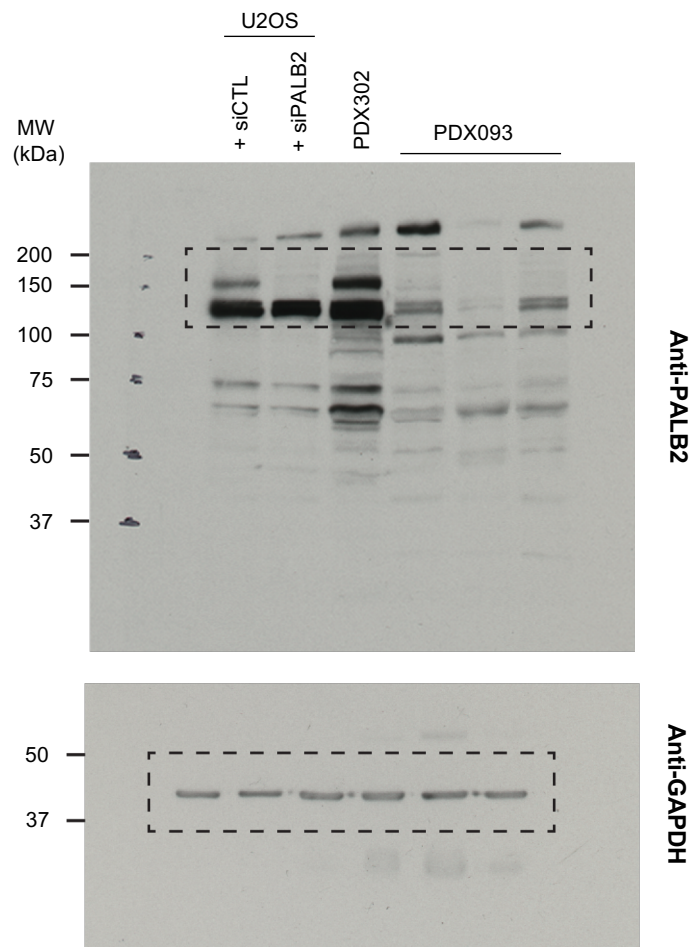

Figure 2D

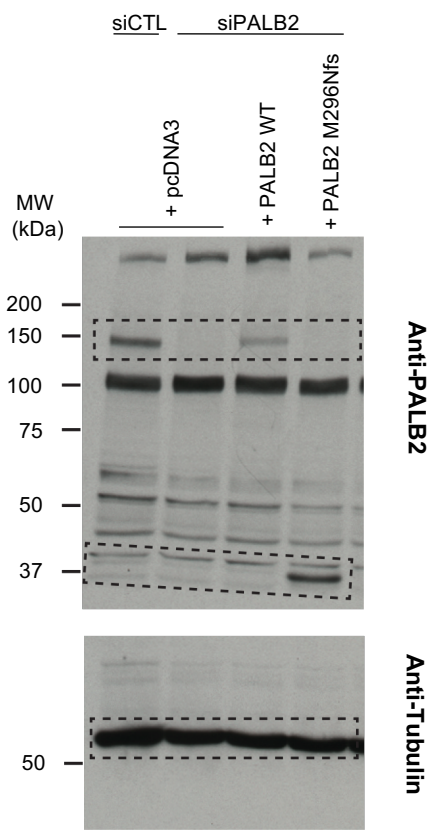

Figure 2E

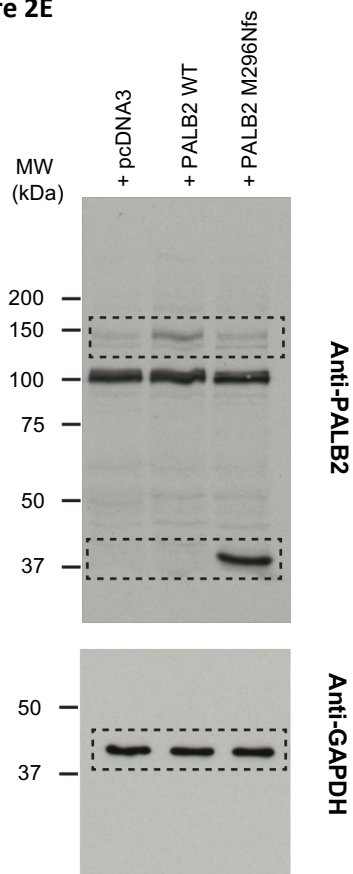

Supplement: Supplementary file 3 — Source Data for Figure 2 [file EMMM-10-e9172-s009.pdf]
