# Supplementary material for: A RAD51 assay feasible in routine tumor samples calls PARP inhibitor response beyond BRCA mutation
Source: EMBO Mol Med. 2018 Oct 30;10(12):e9172. doi: 10.15252/emmm.201809172 (PMC6284440; doi:10.15252/emmm.201809172)
Supplement: Supplementary file 5 — Table EV1 [file EMMM-10-e9172-s003.docx]

**Table EV1: Descriptive analysis from available patients of PDX cohort-1.**

| **Variable** | **N** | **Mean** | **Median (Min-Max)** | **Levels** | **n** | **%** | **Positive** | **Negative** | **Result** | **YES** | **NO** |
| --- | --- | --- | --- | --- | --- | --- | --- | --- | --- | --- | --- |
| **Median age at cancer diagnosis**  **(in years)** | 13 | 57.85 | 58.19 (31.59-93.53) | - | - | - | - | - | - | - | - |
| **Median time between diagnosis and first relapse**  **(in months)** | 11 | 20.27 | 15 (5-96) | - | - | - | - | - | - | - | - |
| **Median time between PDX and diagnosis**  **(in months)** | 13 | 18.92 | 11 (1-97) | - | - | - | - | - | - | - | - |
| **Median time between PDX and first relapse**  **(in months)** | 8 | 6.88 | 6 (0-15) | - | - | - | - | - | - | - | - |
| **Stage at diagnosis (n=13)** | - | - | - | Stage I | 2 | 15.39 | - | - | - | - | - |
|  | - | - | - | Stage II | 7 | 53.85 | - | - | - | - | - |
|  | - | - | - | Stage III | 3 | 23.08 | - | - | - | - | - |
|  | - | - | - | Stage IV | 1 | 7.69 | - | - | - | - | - |
| **IHC characterization (n=13)** | - | - | - | Estrogen receptor | - | - | 4 | 9 | - | - | - |
|  | - | - | - | Progesterone receptor | - | - | 2 | 11 | - | - | - |
|  | - | - | - | Her2 status | - | - | 0 | 13 | - | - | - |
| **Cancer subtype (n=13)** | - | - | - | Luminal A | 1 | - | - | - | - | - | - |
|  | - | - | - | Luminal B | 4 | - | - | - | - | - | - |
|  | - | - | - | TNBC | 8 | - | - | - | - | - | - |
| **Mutational panel assessed (n=13)** | - | - | - | Mutation | 1 | - | - | - | TP53 Y106X | - | - |
|  | - | - | - | WT | 1 | - | - | - | NA | - | - |
|  | - | - | - | Not assessed | 11 | - | - | - | NA | - | - |
| **Patient's therapy exposure previous to PDX (n=13)** | - | - | - | Anthracyclines | - | - | - | - | - | 8 | 5 |
|  | - | - | - | Taxanes | - | - | - | - | - | 9 | 4 |
|  | - | - | - | Platinum | - | - | - | - | - | 4 | 9 |
|  | - | - | - | Capecitabine | - | - | - | - | - | 3 | 10 |
|  | - | - | - | Gemcitabine | - | - | - | - | - | 3 | 10 |
|  | - | - | - | Hormone therapy | - | - | - | - | - | 1 | 12 |
|  | - | - | - | Eribuline | - | - | - | - | - | 1 | 12 |
|  | - | - | - | Other therapies | - | - | - | - | - | 4 | 9 |

Comments: Median time between diagnosis and first relapse (in years) N=11 because 2 patients had no relapse.

Median time between PDX and first relapse (in months) n=8 because 2 patients had no relapse and in 3 cases PDX sample was collected previous to first relapse.
