# Supplementary material for: A RAD51 assay feasible in routine tumor samples calls PARP inhibitor response beyond BRCA mutation
Source: EMBO Mol Med. 2018 Oct 30;10(12):e9172. doi: 10.15252/emmm.201809172 (PMC6284440; doi:10.15252/emmm.201809172)
Supplement: Supplementary file 6 — Table EV2 [file EMMM-10-e9172-s004.docx]

**Table EV2. Exome sequencing results of HRR-related genes in PDX cohort-1.**

| **SAMPLE ID** | **GENE** | **SV-PROTEIN-CHANGE** | **SV-CDS-CHANGE** | **SOMATIC STATUS/ FUNCTIONAL IMPACT** | **VARIANT-TYPE** | **SV-GENOME- POSITION** | **SV-PERCENT- READS** | **CNA-EXONS** | **CNA-RATIO** | **CNA-TYPE** | **known-Other** |
| --- | --- | --- | --- | --- | --- | --- | --- | --- | --- | --- | --- |
| **PDX44** | ***PTEN*** | C211fs | c.631dupT | likely | short-variant | chr10:89712011 | 100 | - | - | - | Trunc/FS |
|  | ***TP53*** | V272_R273delinsG | c.815_817delTGC | unknown | short-variant | chr17:7577120 | 98,6 | - | - | - | unknown-InDel |
| **PDX060** | ***CCNE1*** | - | - | NA | CNA | - | - | 11 of 11 | 1,97 | amplification | amplification |
|  | ***MSH6*** | D763H | c.2287G>C | unknown | short-variant | chr2:48027409 | 33,3 | - | - | - | unknown-Missense |
|  | ***PMS2*** | G857A | c.2570G>C | likely | short-variant | chr7:6013049 | 33,9 | - | - | - | known-Missense |
|  | ***POLD2*** | L132M | c.394C>A | unknown | short-variant | chr7:44157595 | 45 | - | - | - | unknown-Missense |
|  | ***PTEN*** | - | - | NA | CNA | - | - | 7 of 9 | -8,29 | loss | Deletion |
|  | ***RAD50*** | - | - | NA | CNA | - | - | 1 of 25 | -3,11 | loss | Deletion |
|  | ***SHPRH*** | R878* | c.2632C>T | likely | short-variant | chr6:146256515 | 72 | - | - | - | Trunc/FS |
|  | ***TDG*** | splice | c.408+1_409-1del | likely | short-variant | chr12:104373850 | 8,8 | - | - | - | Splice |
|  | ***TP53*** | R306* | c.916C>T | known | short-variant | chr17:7577022 | 100 | - | - | - | Trunc/FS |
| **PDX093** | ***FAM175B*** | - | - | NA | CNA | - | - | 1 of 9 | -5,94 | loss | Deletion |
|  | ***PALB2*** | M296Nfs | c.886dupA | likely | short-variant | chr16:23646980 | 44,2 | - | - | - | Trunc/FS |
|  | ***PMS2*** | G857A | c.2570G>C | likely | short-variant | chr7:6013049 | 41,7 | - | - | - | known-Missense |
|  | ***POLB*** | V177_R182delinsG | c.530_544delTCTGTGGCAGTTTCA | unknown | short-variant | chr8:42214889 | 100 | - | - | - | unknown-InDel |
|  | ***PTEN*** | splice | c.209+2delT | likely | short-variant | chr10:89685315 | 100 | - | - | - | Splice |
|  | ***RAD50*** | R365Q | c.1094G>A | unknown | short-variant | chr5:131924421 | 92,6 | - | - | - | unknown-Missense |
|  | ***RIF1*** | G389C | c.1165G>T | unknown | short-variant | chr2:152292064 | 52,3 | - | - | - | unknown-Missense |
|  | ***RTEL1*** | A435T | c.1303G>A | unknown | short-variant | chr20:62317180 | 49,3 | - | - | - | unknown-Missense |
|  | ***TDG*** | splice | c.23+1_24-1del | likely | short-variant | chr12:104359838 | 9 | - | - | - | Splice |
| **PDX093OR** | ***FAM175B*** | - | - | NA | CNA | - | - | 1 of 9 | -10 | loss | Deletion |
|  | ***FANCI*** | - | - | NA | CNA | - | - | 37 of 37 | 1,49 | amplification | amplification |
|  | ***PMS2*** | G857A | c.2570G>C | likely | short-variant | chr7:6013049 | 52,2 | - | - | - | known-Missense |
|  | ***POLB*** | V177_R182delinsG | c.530_544delTCTGTGGCAGTTTCA | unknown | short-variant | chr8:42214889 | 32,1 | - | - | - | unknown-InDel |
|  | ***POLG*** | - | - | NA | CNA | - | - | 22 of 22 | 1,46 | amplification | amplification |
|  | ***PTEN*** | splice | c.209+2delT | likely | short-variant | chr10:89685315 | 98 | - | - | - | Splice |
|  | ***RAD50*** | R365Q | c.1094G>A | unknown | short-variant | chr5:131924421 | 97,6 | - | - | - | unknown-Missense |
|  | ***RIF1*** | G389C | c.1165G>T | unknown | short-variant | chr2:152292064 | 54,4 | - | - | - | unknown-Missense |
|  | ***RTEL1*** | A435T | c.1303G>A | unknown | short-variant | chr20:62317180 | 44,9 | - | - | - | unknown-Missense |
| **PDX094** | ***ATM*** | E2294D | c.6882A>T | unknown | short-variant | chr11:108196859 | 99,3 | - | - | - | unknown-Missense |
|  | ***BLM*** | F194fs | c.581_582delTT | known | short-variant | chr15:91293074 | 30,2 | - | - | - | Trunc/FS |
|  | ***DDX11*** | E201K | c.601G>A | unknown | short-variant | chr12:31238023 | 8,5 | - | - | - | unknown-Missense |
|  | ***FANCL*** | I40V | c.118A>G | unknown | short-variant | chr2:58459226 | 32,6 | - | - | - | unknown-Missense |
|  | ***GTF2H3*** | - | - | NA | CNA | - | - | 13 of 13 | 1,52 | amplification | amplification |
|  | ***MAD2L1*** | - | - | NA | CNA | - | - | 5 of 5 | 3,35 | amplification | amplification |
|  | ***PARP2*** | E350D | c.1050G>C | unknown | short-variant | chr14:20824100 | 39,8 | - | - | - | unknown-Missense |
|  | ***PMS2*** | G857A | c.2570G>C | likely | short-variant | chr7:6013049 | 40 | - | - | - | known-Missense |
|  | ***TP53*** | G266* | c.796G>T | known | short-variant | chr17:7577142 | 100 | - | - | - | Trunc/FS |
| **PDX098** | ***ATM*** | V1912L | c.5734G>C | unknown | short-variant | chr11:108178683 | 100 | - | - | - | unknown-Missense |
|  | ***DNTTIP2*** | T640fs | c.1918dupA | likely | short-variant | chr1:94337776 | 37 | - | - | - | Trunc/FS |
|  | ***MPG*** | R246C | c.736C>T | unknown | short-variant | chr16:135615 | 26 | - | - | - | unknown-Missense |
|  | ***PALB2*** | V78I | c.232G>A | unknown | short-variant | chr16:23647635 | 34,2 | - | - | - | unknown-Missense |
|  | ***TP53*** | R249S | c.747G>T | known | short-variant | chr17:7577534 | 100 | - | - | - | known-Missense |
|  | ***XRCC5*** | M427I | c.1281G>A | unknown | short-variant | chr2:217002841 | 36,2 | - | - | - | unknown-Missense |
|  | ***ZW10*** | D615N | c.1843G>A | unknown | short-variant | chr11:113609027 | 100 | - | - | - | unknown-Missense |
| **PDX102** | ***FANCB*** | - | - | NA | CNA | - | - | 8 of 8 | 1,45 | amplification | amplification |
|  | ***POLA1*** | - | - | NA | CNA | - | - | 37 of 37 | 1,47 | amplification | amplification |
|  | ***RAD54B*** | K721I | c.2162A>T | unknown | short-variant | chr8:95392458 | 10,9 | - | - | - | unknown-Missense |
|  | ***TOP3B*** | S767P | c.2299T>C | unknown | short-variant | chr22:22311776 | 8,9 | - | - | - | unknown-Missense |
|  | ***TP53*** | Q331H | c.993G>C | known | short-variant | chr17:7576853 | 99 | - | - | - | known-Missense |
| **PDX137** | ***ATR*** | A901P | c.2701G>C | unknown | short-variant | chr3:142272173 | 14,9 | - | - | - | unknown-Missense |
|  | ***ERCC2*** | R616P | c.1847G>C | unknown | short-variant | chr19:45856059 | 65,8 | - | - | - | unknown-Missense |
|  | ***PARP4*** | V458I | c.1372G>A | unknown | short-variant | chr13:25058867 | 15,2 | - | - | - | unknown-Missense |
|  | ***PMS2*** | G857A | c.2570G>C | likely | short-variant | chr7:6013049 | 69,2 | - | - | - | known-Missense |
|  | ***POLB*** | L11fs | c.32_33delTC | likely | short-variant | chr8:42196170 | 44,7 | - | - | - | Trunc/FS |
|  | ***REV1*** | - | - | NA | CNA | - | - | 22 of 22 | 1,48 | amplification | amplification |
|  | ***TP53*** | S166* | c.497C>G | known | short-variant | chr17:7578433 | 100 | - | - | - | Trunc/FS |
| **STG139** | ***CUL4A*** | - | - | NA | CNA | - | - | 20 of 20 | 1,82 | amplification | amplification |
|  | ***ERCC5*** | - | - | NA | CNA | - | - | 15 of 15 | 1,77 | amplification | amplification |
|  | ***GTF2H2*** | - | - | NA | CNA | - | - | 1 of 15 | 3,05 | amplification | amplification |
|  | ***LIG4*** | - | - | NA | CNA | - | - | 1 of 1 | 1,7 | amplification | amplification |
|  | ***PER1*** | - | - | NA | CNA | - | - | 22 of 22 | 1,5 | amplification | amplification |
|  | ***PMS2*** | G857A | c.2570G>C | likely | short-variant | chr7:6013049 | 77,5 | - | - | - | known-Missense |
|  | ***RNASEH2A*** | - | - | NA | CNA | - | - | 8 of 8 | 1,96 | amplification | amplification |
|  | ***SHPRH*** | D1673E | c.5019C>G | unknown | short-variant | chr6:146207860 | 39,9 | - | - | - | unknown-Missense |
|  | ***SLX4*** | P763S | c.2287C>T | unknown | short-variant | chr16:3642740 | 42 | - | - | - | unknown-Missense |
|  | ***SLX4*** | P763S | c.2287C>T | unknown | short-variant | chr16:3642740 | 42 | - | - | - | unknown-Missense |
|  | ***SMARCA4*** | - | - | NA | CNA | - | - | 17 of 34 | 2,34 | amplification | amplification |
|  | ***TP53*** | E258* | c.772G>T | known | short-variant | chr17:7577509 | 99,2 | - | - | - | Trunc/FS |
|  | ***TREX2*** | G169V | c.506G>T | unknown | short-variant | chrX:152710383 | 26,8 | - | - | - | unknown-Missense |
| **PDX156** | ***MSH3*** | A57P | c.169G>C | unknown | short-variant | chr5:79950715 | 21,7 | - | - | - | unknown-Missense |
|  | ***POLB*** | - | - | NA | CNA | - | - | 14 of 14 | 1,48 | amplification | amplification |
|  | ***POLG*** | Q53dup | c.156_158dupGCA | unknown | short-variant | chr15:89876827 | 74,4 | - | - | - | unknown-Other |
|  | ***TOP3B*** | S767P | c.2299T>C | unknown | short-variant | chr22:22311776 | 8 | - | - | - | unknown-Missense |
|  | ***TP53*** | R280K | c.839G>A | known | short-variant | chr17:7577099 | 100 | - | - | - | known-Missense |
| **PDX197** | ***CLK2*** | Y430C | c.1289A>G | unknown | short-variant | chr1:155233769 | 48,3 | - | - | - | unknown-Missense |
|  | ***CUL4A*** | - | - | NA | CNA | - | - | 20 of 20 | 1,68 | amplification | amplification |
|  | ***ERCC5*** | - | - | NA | CNA | - | - | 15 of 15 | 1,75 | amplification | amplification |
|  | ***GTF2H1*** | K60T | c.179A>C | unknown | short-variant | chr11:18357325 | 26,6 | - | - | - | unknown-Missense |
|  | ***LIG1*** | R643H | c.1928G>A | unknown | short-variant | chr19:48631171 | 94,6 | - | - | - | unknown-Missense |
|  | ***LIG4*** | G120E | c.359G>A | unknown | short-variant | chr13:108863258 | 10,6 | - | - | - | unknown-Missense |
|  | ***MSH2*** | N596S | c.1787A>G | unknown | short-variant | chr2:47702191 | 98,2 | - | - | - | unknown-Missense |
|  | ***MSH3*** | A57_A65del | c.169_195delGCCGCAGCGGCCGCAGCGCCCCCAGCG | unknown | short-variant | chr5:79950709 | 10,1 | - | - | - | unknown-InDel |
|  | ***NBN*** | K223E | c.667A>G | unknown | short-variant | chr8:90983436 | 17,1 | - | - | - | unknown-Missense |
|  | ***PTEN*** | S229* | c.686C>G | likely | short-variant | chr10:89717661 | 100 | - | - | - | Trunc/FS |
|  | ***RECQL5*** | A757P | c.2269G>C | unknown | short-variant | chr17:73625234 | 37,7 | - | - | - | unknown-Missense |
|  | ***TP53*** | R175H | c.524G>A | known | short-variant | chr17:7578406 | 98,4 | - | - | - | known-Missense |
| **STG201** | ***FANCD2*** | S64fs | c.192_195delTCAG | likely | short-variant | chr3:10074640 | 98,1 | - | - | - | Trunc/FS |
|  | ***GTF2H4*** | - | - | NA | CNA | - | - | 6 of 13 | -3,2 | loss | Deletion |
|  | ***POLG*** | Q53dup | c.156_158dupGCA | unknown | short-variant | chr15:89876827 | 79 | - | - | - | unknown-Other |
|  | ***POLH*** | S512fs | c.1535_1559delCACCATCCAAGCCCTCATTACCTTT | likely | short-variant | chr6:43581684 | 95 | - | - | - | Trunc/FS |
|  | ***PTEN*** | G165fs | c.495_516delAGTAACTATTCCCAGTCAGAGG | likely | short-variant | chr10:89711874 | 100 | - | - | - | Trunc/FS |
|  | ***TP53*** | M237I | c.711G>A | known | short-variant | chr17:7577570 | 89,5 | - | - | - | known-Missense |
| **STG201OR** | ***FANCD2*** | S64fs | c.192_195delTCAG | likely | short-variant | chr3:10074640 | 100 | - | - | - | Trunc/FS |
|  | ***POLD1*** | V866V | c.2598G>C | unknown | short-variant | chr19:50918203 | 52,97 | - | - | - | unknown-Other |
|  | ***POLG*** | Q53dup | c.156_158dupGCA | unknown | short-variant | chr15:89876827 | 77,64 | - | - | - | unknown-Other |
|  | ***POLH*** | S512fs | c.1535_1559delCACCATCCAAGCCCTCATTACCTTT | likely | short-variant | chr6:43581684 | 100 | - | - | - | Trunc/FS |
|  | ***PTEN*** | V166fs | c.495_516delAGTAACTATTCCCAGTCAGAGG | likely | short-variant | chr10:89711874 | 94,74 | - | - | - | Trunc/FS |
|  | ***RAD17*** | K535fs | c.1605_1605+2delGGT | likely | short-variant | chr5:68692372 | 38,89 | - | - | - | Trunc/FS |
|  | ***RECQL5*** | splice | c.1586-3_1586-2dupCA | likely | short-variant | chr17:73626918 | 73,24 | - | - | - | Splice |
|  | ***TDG*** | - | c.-78402696_793-7del | unknown | short-variant | chr12:25957119 | 19,83 | - | - | - | unknown-Other |
|  | ***TP53*** | M237I | c.711G>A | known | short-variant | chr17:7577570 | 100 | - | - | - | known-Missense |
| **PDX270** | ***CHAF1A*** | G197V | c.590G>T | unknown | short-variant | chr19:4409386 | 39,7 | - | - | - | unknown-Missense |
|  | ***RAD54L*** | V516fs | c.1546delG | likely | short-variant | chr1:46739354 | 100 | - | - | - | Trunc/FS |
|  | ***RECQL5*** | K931N | c.2793G>C | unknown | short-variant | chr17:73624310 | 7,9 | - | - | - | unknown-Missense |
|  | ***SMC1B*** | R1210P | c.3629G>C | unknown | short-variant | chr22:45740516 | 31,4 | - | - | - | unknown-Missense |
|  | ***TP53*** | S241A | c.721T>G | known | short-variant | chr17:7577560 | 95,7 | - | - | - | known-Missense |
|  | ***TP73*** | T188A | c.562A>G | unknown | short-variant | chr1:3638717 | 8,8 | - | - | - | unknown-Missense |
| **PDX288** | ***ERCC5*** | S1097C | c.3290C>G | unknown | short-variant | chr13:103527982 | 23,2 | - | - | - | unknown-Missense |
|  | ***POLB*** | - | - | NA | CNA | - | - | 14 of 14 | 1,59 | amplification | amplification |
|  | ***TP53*** | splice | c.993+1G>A | known | short-variant | chr17:7576852 | 98,9 | - | - | - | Splice |
| **PDX291** | ***RECQL5*** | splice | c.1586-3_1586-2dupCA | likely | short-variant | chr17:73626918 | 46,89 | - | - | - | Splice |
|  | ***SMARCAL1*** | C562F | c.1685G>T | unknown | short-variant | chr2:217303183 | 7,59 | - | - | - | unknown-Missense |
|  | ***TDG*** | - | c.-78402712_793-5del | unknown | short-variant | chr12:25957103 | 76,6 | - | - | - | unknown-Other |
|  | ***TP53*** | Y107fs | c.321delC | known | short-variant | chr17:7579365 | 99,05 | - | - | - | Trunc/FS |
|  | ***UNG*** | P172T | c.514C>A | unknown | short-variant | chr12:109539785 | 45,05 | - | - | - | unknown-Missense |
| **PDX302** | ***CLSPN*** | E43D | c.129A>C | unknown | short-variant | chr1:36230823 | 22,06 | - | - | - | unknown-Missense |
|  | ***DCLRE1A*** | R138* | c.412C>T | likely | short-variant | chr10:115612530 | 100 | - | - | - | Trunc/FS |
|  | ***DDB2*** | splice | c.1024-5_1077delCCTAGGCAGCCTGGCATCCTCGCTACAACCTCATTGTTGTGGGCCGATACCCAGATCCT | likely | short-variant | chr11:47259380 | 17,54 | - | - | - | Splice |
|  | ***RAD17*** | splice | c.1605_1605+2delGGTinsAAA | likely | short-variant | chr5:68692373 | 13,51 | - | - | - | Splice |
|  | ***REV1*** | A1090_S1091insSA | c.3264_3269dupCAGCGC | unknown | short-variant | chr2:100020162 | 52,05 | - | - | - | unknown-InDel |
|  | ***TOP1MT*** | A88V | c.263C>T | unknown | short-variant | chr8:144411617 | 100 | - | - | - | unknown-Missense |
|  | ***TP53*** | T256fs | c.766dupA | known | short-variant | chr17:7577514 | 100 | - | - | - | Trunc/FS |
| **PDX302OR** | ***CLSPN*** | E43D | c.129A>C | unknown | short-variant | chr1:36230823 | 30 | - | - | - | unknown-Missense |
|  | ***DCLRE1A*** | R138* | c.412C>T | likely | short-variant | chr10:115612530 | 100 | - | - | - | Trunc/FS |
|  | ***DDB2*** | splice | c.1024-5_1077delCCTAGGCAGCCTGGCATCCTCGCTACAACCTCATTGTTGTGGGCCGATACCCAGATCCT | likely | short-variant | chr11:47259380 | 37,5 | - | - | - | Splice |
|  | ***REV1*** | A1090_S1091insSA | c.3264_3269dupCAGCGC | unknown | short-variant | chr2:100020162 | 46,58 | - | - | - | unknown-InDel |
|  | ***TOP1MT*** | A88V | c.263C>T | unknown | short-variant | chr8:144411617 | 100 | - | - | - | unknown-Missense |
|  | ***TP53*** | T256fs | c.766dupA | known | short-variant | chr17:7577514 | 100 | - | - | - | Trunc/FS |

*CNA: copy-number-alteration
