# Supplementary material for: A RAD51 assay feasible in routine tumor samples calls PARP inhibitor response beyond BRCA mutation
Source: EMBO Mol Med. 2018 Oct 30;10(12):e9172. doi: 10.15252/emmm.201809172 (PMC6284440; doi:10.15252/emmm.201809172)
Supplement: Supplementary file 7 — Table EV3 [file EMMM-10-e9172-s005.docx]

**Table EV3. Sequencing results of PDX cohort-2.**

| **SAMPLE ID** | **GENE** | **SV-PROTEIN-CHANGE** | **SV-CDS-CHANGE** | **SOMATIC STATUS/ FUNCTIONAL IMPACT** | **HOMOZYGOSITY** |
| --- | --- | --- | --- | --- | --- |
| **HBCx1** | ***TP53*** | L188 fs35aaTer |  | known | homozygous |
| **HBCx2** | ***AKT1*** | E17K |  | known | homozygous |
|  | ***RB1*** | del from ex18 |  | known |  |
|  | ***TP53*** | A276D |  | known | homozygous |
| **HBCx3** | ***PTEN*** | P246 fs8aa |  | known | homozygous |
|  | ***TP53*** | Q144Ter |  | known | homozygous |
| **HBCx6** | ***NF1*** | S1030Ter |  | new | heterozygous |
|  | ***RB1*** | del ex1-17 |  | known |  |
|  | ***TP53*** | T102 fs19aaTer |  | known | homozygous |
| **HBCx7** | ***ARID1A*** | M1673Ter |  | new | heterozygous |
|  | ***ATM*** | D1853N |  | known | heterozygous |
|  | ***CDKN2A*** | - |  | deletion |  |
|  | ***EP300*** | D1713Ter |  | new | heterozygous |
|  | ***KDR*** | chr4-55976819-A->G-(spliceSite) |  | new | heterozygous |
|  | ***MLL3*** | Q2054Ter |  | new |  |
|  | ***ROS1*** | Q1369Ter |  | new | homozygous |
| **HBCx8** | ***BRCA1*** | Q81* | c.241C>T | likely | homozygous |
|  | ***NRAS*** | Q61K |  | known | heterozygous |
| **HBCx9** | ***ATM*** | Q1128R |  | new | homozygous |
|  | ***CDH1*** | A617T |  | known | heterozygous |
|  | ***TP53*** | V143 fs25aaTer |  | known | homozygous |
| **HBCx10** | ***BRCA2*** | Q3036* | c.9106C>T | likely | homozygous |
|  | ***PTEN*** | del ex3 |  | known |  |
|  | ***RB1*** | deleted gen |  | known |  |
|  | ***TP53*** | V157F |  | known | homozygous |
| **HBCx11** | ***BRCA1*** | K654Sfs*47 | c.1961del | likely | homozygous |
|  | ***ARID1A*** | E1924 Ter |  | known | heterozygous |
|  | ***KDM6A*** | P807fs 26aa (ins 19nt) |  | new | heterozygous |
|  | ***MLL2*** | E2186 fs73aa (del 16 nt) |  | new | heterozygous |
|  | ***PI3KR1*** | del P568-D569-L570-I571 |  | known | homozygous |
|  | ***STK11*** | F354L |  | known | homozygous |
|  | ***TP53*** | R249 fs96aaTer |  | known | homozygous |
| **HBCx12b** | ***NF1*** | V1764fs 9aa |  | new | heterozygous |
|  | ***TP53*** | del G108-G109 |  | known | homozygous |
| **HBCx14** | ***HRAS*** | E98Ter |  | new | heterozygous |
|  | ***NOTCH1*** | loss |  | known |  |
|  | ***RB1*** | del ex1 |  | known |  |
|  | ***TP53*** | Y163C |  | known | homozygous |
| **HBCx15** | ***KIT*** | T594I |  | known | heterozygous |
|  | ***PITCH1*** | D39H |  | known | heterozygous |
|  | ***TP53*** | P151H |  | known | homozygous |
| **HBCx16** | ***PTEN*** | del ex3-4-5 |  | known |  |
|  | ***TP53*** | E180 ins in frame 6aa (GAAPTM) |  | known | homozygous |
| **HBCx17** | ***BRCA2*** | S2012Qfs*5 | c.6033_6034del | likely | homozygous |
|  | ***AKT1*** | D46E |  | known | homozygous |
|  | ***CDKN2A*** | deleted gene |  | known |  |
|  | ***KDM6A*** | partially deleted gene |  | known |  |
|  | ***TP53*** | H193 fs53aaTer |  | known | homozygous |
| **HBCx23** | ***CDKN2A*** | deleted gene |  | known |  |
|  | ***TP53*** | del N131 |  | known | homozygous |
| **HBCx24** | ***TP53*** | K292 fs11aaTer |  | known | homozygous |
| **HBCx27** | ***ATM*** | D1853N |  | known | heterozygous |
|  | ***CDKN2A*** | deleted gene |  | known |  |
|  | ***KDM6A*** | Q1248Ter |  | new | homozygous |
|  | ***TP53*** | V274D |  | known | homozygous |
| **HBCx28** | ***BRCA1*** | F46_R71del;C64* | c.212+3A>G | likely | homozygous |
|  | ***PTEN*** | loss |  | known |  |
|  | ***TP53*** | R175H |  | known | homozygous |
| **HBCx30** | ***CDKN2A*** | deleted gene |  | known |  |
|  | ***PTEN*** | loss |  | known |  |
|  | ***TP53*** | F134L |  | known | homozygous |
| **HBCx31** | ***AKT1*** | E17K |  | known | homozygous |
|  | ***TP53*** | R175H |  | known | homozygous |
| **HBCx33** | ***ATRX*** | M1800K |  | known | heterozygous |
|  | ***TP53*** | V218E |  | known | homozygous |
| **HBCx39** | ***TP53*** | Y220C |  | known | homozygous |
| **T168** | ***BRCA1*** | S1524Lfs*24 | c.4570del | likely | homozygous |
|  | ***TP53*** | R196Ter |  | known | homozygous |
| **T174** | ***CDKN2A*** | deleted gene |  | known |  |
|  | ***PIK3CA*** | H1047Q |  | known | homozygous |
|  | ***TP53*** | T256P |  | known | homozygous |
| **T180R** | ***CREBBP*** | deleted gene |  | known |  |
|  | ***TP53*** | R175H |  | known | heterozygous |
|  | ***TP53*** | L130 fs38 |  | new | heterozygous |
|  | ***TSC1*** | deleted exons 1 to 5 |  | known |  |
| **T298** | ***PALB2*** | S475* | c.1424C>G | likely | homozygous |
|  | ***CREBBP*** | P1096 fs13aa (TACAGTGCTTCTAGGGTTGG/C) |  | new | heterozygous |
|  | ***NOTCH1*** | E1447 inframe del 116aa |  | new | homozygous |
|  | ***PIK3CA*** | H1047Q |  | known | homozygous |
| **T311R** | ***ATM*** | N1739R |  | known | heterozygous |
|  | ***MLL3*** | R2139Ter |  | new | heterozygous |
|  | ***NF1*** | Q83Ter |  | known | homozygous |
|  | ***PIK3CA*** | H1047Q |  | known | homozygous |
|  | ***TP53*** | D259V |  | known | homozygous |
| **T330** | ***BRCA1*** | S1280* | c.3839_3844delins5 | likely | homozygous |
|  | ***NRAS*** | P185S |  | known | heterozygous |
|  | ***TP53*** | E204Ter |  | known | homozygous |
| **T381** | ***TP53*** | chr17-7578370-C->T/T-(spliceSite) |  | known |  |
