# Supplementary material for: A RAD51 assay feasible in routine tumor samples calls PARP inhibitor response beyond BRCA mutation
Source: EMBO Mol Med. 2018 Oct 30;10(12):e9172. doi: 10.15252/emmm.201809172 (PMC6284440; doi:10.15252/emmm.201809172)
Supplement: Supplementary file 8 — Table EV4 [file EMMM-10-e9172-s006.docx]

**Table EV4. Characteristics of HBOC patients’ tumors.**

| **Patient** | **Sample** | **PALB2 ALTERATION** | **TYPE OF SAMPLE** | **DIAGNOSIS** | **AGE OF DIAGNOSIS** | **H.GRADE** | **ER STATUS** | **PR STATUS** | **Her2 STATUS** | **Ki67 (%)** |
| --- | --- | --- | --- | --- | --- | --- | --- | --- | --- | --- |
| 01 | 01 | - | Breast surgery | IDC | 58 | 3 | + | + | - | 20 |
| 02 | 02 | - | Tumorectomy | IDC | 29 | 3 | - | - | - | 90 |
| 03 | 03 | - | Core biopsy | IDC | 40 | 3 | - | - | - | 70, 80 |
| 04 | 04 | - | Core biopsy | IDC | 48 | 2 | - | - | - | 50 |
| 05 | 05 | - | Core biopsy | IDC | 52 | 1 | + | + | - | 8 |
| 06 | 06 | - | Core biopsy | IDC | 31 | 2 | + | - | - | 30 |
| 07 | 07 | - | Core biopsy | IDC | 27 | 3 | - | + | - | 95 |
| 08 | 08 | - | Core biopsy | Invasive carcinoma | 31 | 3 | - | - | - | 70 |
| 09 | 09 | - | Core biopsy | IDC | 35 | 2 | + | + | + | 15 |
| 10 | 10 | - | Breast surgery | IDC | 28 | 2 | + | + | + | 40 |
| 11 | 11 | - | Core biopsy | IDC | 32 | 3 | - | - | - | 85 |
| 12 | 12 | - | Breast surgery | IDC | 38 | 2 | + | + | - | 35 |
| 13 | 13 | c.3362delG p.(Gly1121Valfs*3) | Breast surgery | IDC | 41 | 3 | + | + | uk | uk |
| 14 | 14 | c.3362delG p.(Gly1121Valfs*3) | Breast surgery | IDC | 42 | 3 | + | - | uk | uk |
| 15 | 15 | c.3201+5G>T Splice | Mastectomy | IDC | 54 | 2 | + | + | - | 22 |
| 16 | 16.1 | c.1675C>T p.(Gln559*) | Core biopsy | IDC | 51 | 3 | + | + | - | 70 |
|  | 16.2 |  | Core biopsy | IDC |  | 3 | + | + | - | 60 |
| 17 | 17 | c.1111G>T p.(Glu371*) | Core biopsy | ILC | 45 | 2 | + | + | - | 24 |
| 18 | 18 | c.2257C>T p.(Arg753*) | Core biopsy | IDC | 46 | 3 | + | + | - | 80 |
| 19 | 19 | c.1240C>T | Core biopsy | IDC | 38 | 3 | - | - | - | 90 |
| 20 | 20.1 | c.3256C>T p.(Arg1086*) | Core biopsy | IDC | 40 | 3 | - | - | - | 50 |
|  | 20.2.1 |  | Core biopsy | IDC |  | 2 | - | - | - | 55 |
|  | 20.2.2 |  | Core biopsy | IDC |  | 2 | + | + | - | 40 |

Rows 13 to 20 correspond with patients with alterations in *PALB2*. The loss-of-function effect of the *PALB2* c.3201+5G>T splicing variant was confirmed by RNA analysis (data not shown). IDC: invasive ductal carcinoma.
