# Supplementary material for: A RAD51 assay feasible in routine tumor samples calls PARP inhibitor response beyond BRCA mutation
Source: EMBO Mol Med. 2018 Oct 30;10(12):e9172. doi: 10.15252/emmm.201809172 (PMC6284440; doi:10.15252/emmm.201809172)
Supplement: Supplementary file 9 — Movie EV1 [file EMMM-10-e9172-s007.zip › MovieEV1/Movie_EV1.docx]

**Movie EV1.** Recruitment of YFP-PALB2 *wild type* to laser-induced DNA damage sites.
